# Supplementary material for: Intratumoral immunotherapy using platelet-cloaked nanoparticles enhances antitumor immunity in solid tumors
Source: Nat Commun. 2021 Mar 31;12:1999. doi: 10.1038/s41467-021-22311-z (PMC8012593; doi:10.1038/s41467-021-22311-z)
Supplement: Supplementary file 1 — Supplementary Information. [file 41467_2021_22311_MOESM1_ESM.pdf]

Supplementary Information:

Intratumoral immunotherapy using platelet-cloaked nanoparticles enhances antitumor immunity in solid tumors

Baharak Bahmani[1,†], Hua Gong[2,†], Brian T. Luk[1,†], Kristofer J. Haushalter[1], Ethel DeTeresa[1], Mark Previti[1], Jiarong Zhou[2], Weiwei Gao[2], Jack D. Bui[3], Liangfang Zhang[2], Ronnie H. Fang[2,*], Jie Zhang[1,*]

[1] Cello Therapeutics, Inc., San Diego, CA 92121

[2] Department of NanoEngineering, University of California San Diego, La Jolla, CA 92093

[3] Department of Pathology, University of California, San Diego, La Jolla, CA 92093

[†]These authors contributed equally to this work.

*Correspondence should be addressed to J.Z. (jie.zhang@cellothera.com) and R.H.F. (rhfang@ucsd.edu)

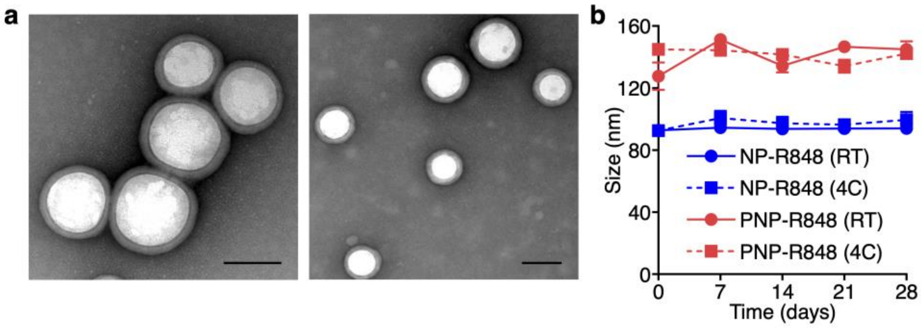

Supplementary Figure 1. Morphology and stability characterization of PNP-R848. a, Transmission electron microscopy visualization (multiple fields of view) of PNP-R848 with uranyl acetate negative staining (scale bars = 100 nm; repeated 3 times). **b**, Size of NP-R848 and PNP-R848 in PBS over 4 weeks at room temperature (RT) or at 4 °C (4C) (n = 3, mean ± SD). Source data are provided as a Source Data file.

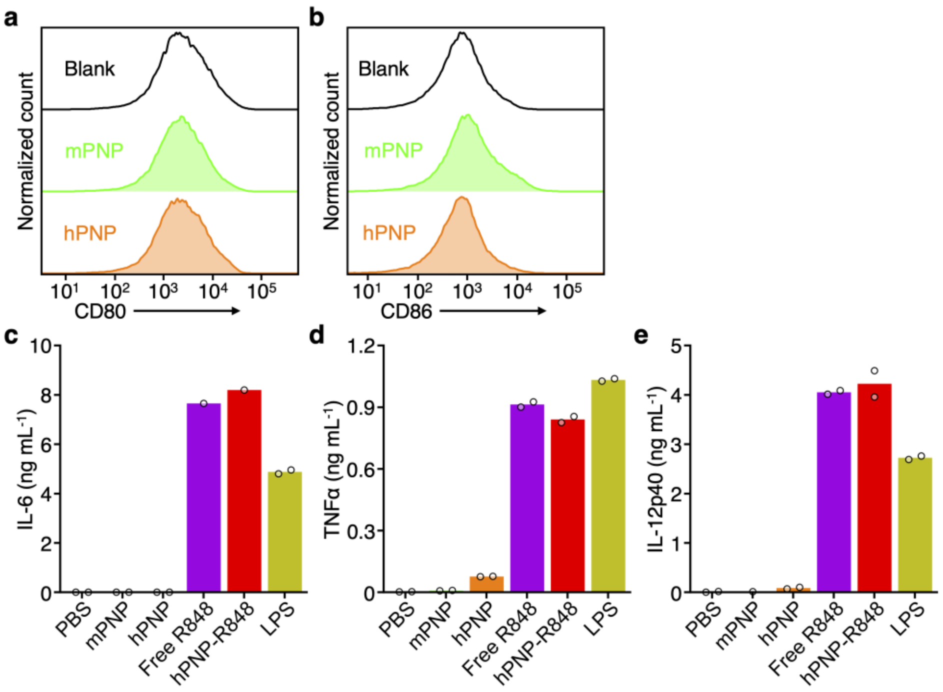

Supplementary Figure 2. In vitro activity of empty PNP. a,b, Expression of CD80 (a) and CD86 (b) by BMDCs after incubation with empty PNP fabricated with mouse platelet membrane (mPNP) or human platelet membrane (hPNP). **c-e**, Secretion of IL-6 (c), TNFα (d), and IL-12p40 (e) by BMDCs after incubation with mPNP, hPNP, free R848, hPNP-R848, or LPS. Source data are provided as a Source Data file.

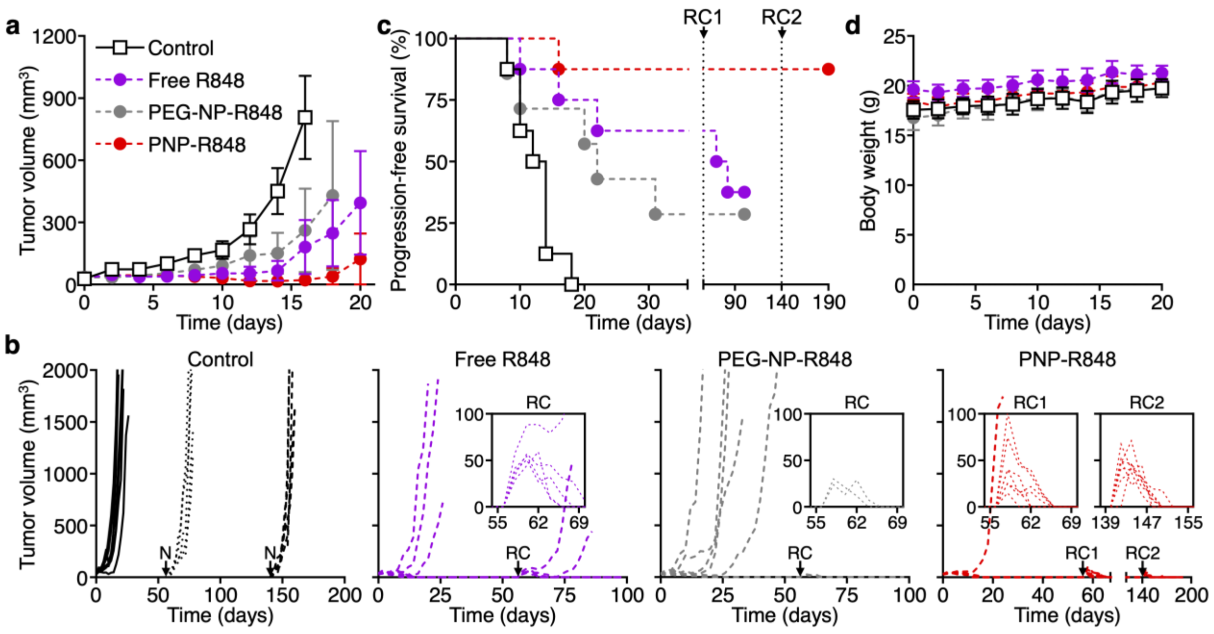

Supplementary Figure 3. Therapeutic antitumor efficacy in an MC38 tumor model at a reduced R848 dosage. a, Average tumor growth kinetics after treatment with a sucrose control (n = 7), free R848 (n = 8), PEG-NP-R848 (n = 7), and PNP-R848 (n = 8) (mean ± SEM). **b**, Individual tumor growth kinetics after treatment with free R848, PEG-NP-R848, and PNP-R848 (N = naïve challenge, RC = re-challenge). The insets depict the growth kinetics after each re-challenge. **c**, Progression-free survival (tumor size < 200 mm^3) of mice after treatment with free R848, PEG-NP-R848, and PNP-R848. **d**, Body weight of mice after treatment with free R848, PEG-NP-R848, and PNP-R848 (mean ± SD). Source data are provided as a Source Data file.

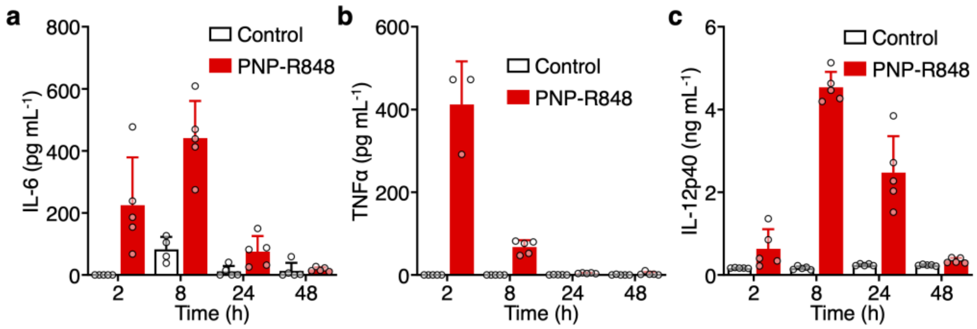

Supplementary Figure 4. Systemic cytokine levels. a-c, Levels of IL-6 (a), TNFα (b), and IL-12p40 (c) in the serum of MC38 tumor-bearing mice after single intratumoral treatment with PNP-R848 (n = 3 for PNP-R848 2 h in b, n = 4 for control 8 h in a, and n = 5 for the rest, mean + SD). Source data are provided as a Source Data file.

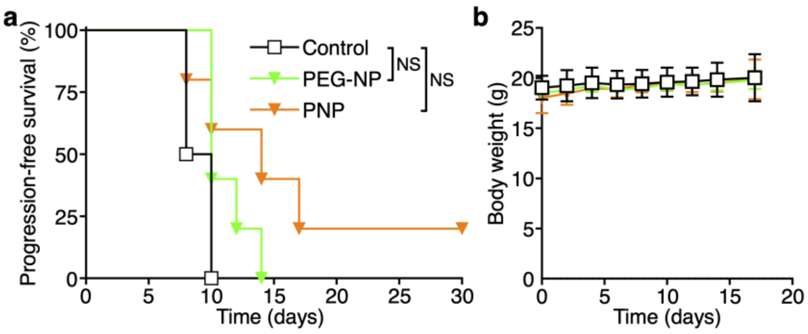

Supplementary Figure 5. Therapeutic antitumor efficacy of empty nanocarriers. a, Progression-free survival (tumor size < 200 mm^3) of MC38 tumor-bearing mice after treatment with PEG-NP or PNP without R848 loading (NS = not significant, two-tailed log-rank test). **b**, Body weight of mice after treatment with PEG-NP or PNP without R848 loading (n = 5, mean + SD). Source data are provided as a Source Data file.

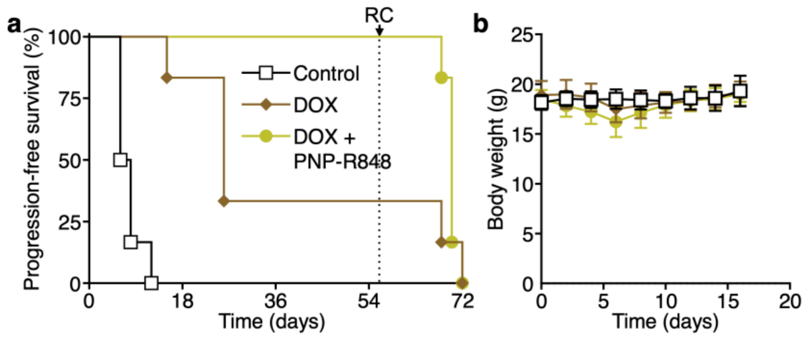

Supplementary Figure 6. Therapeutic efficacy in combination with chemotherapy. a, Progression-free survival (tumor size < 200 mm^3) of mice after treatment with doxorubicin (DOX) or DOX + PNP-R848. **b**, Body weight of mice after treatment with doxorubicin (DOX) or DOX + PNP-R848 (n = 6, mean ± SD). Source data are provided as a Source Data file.

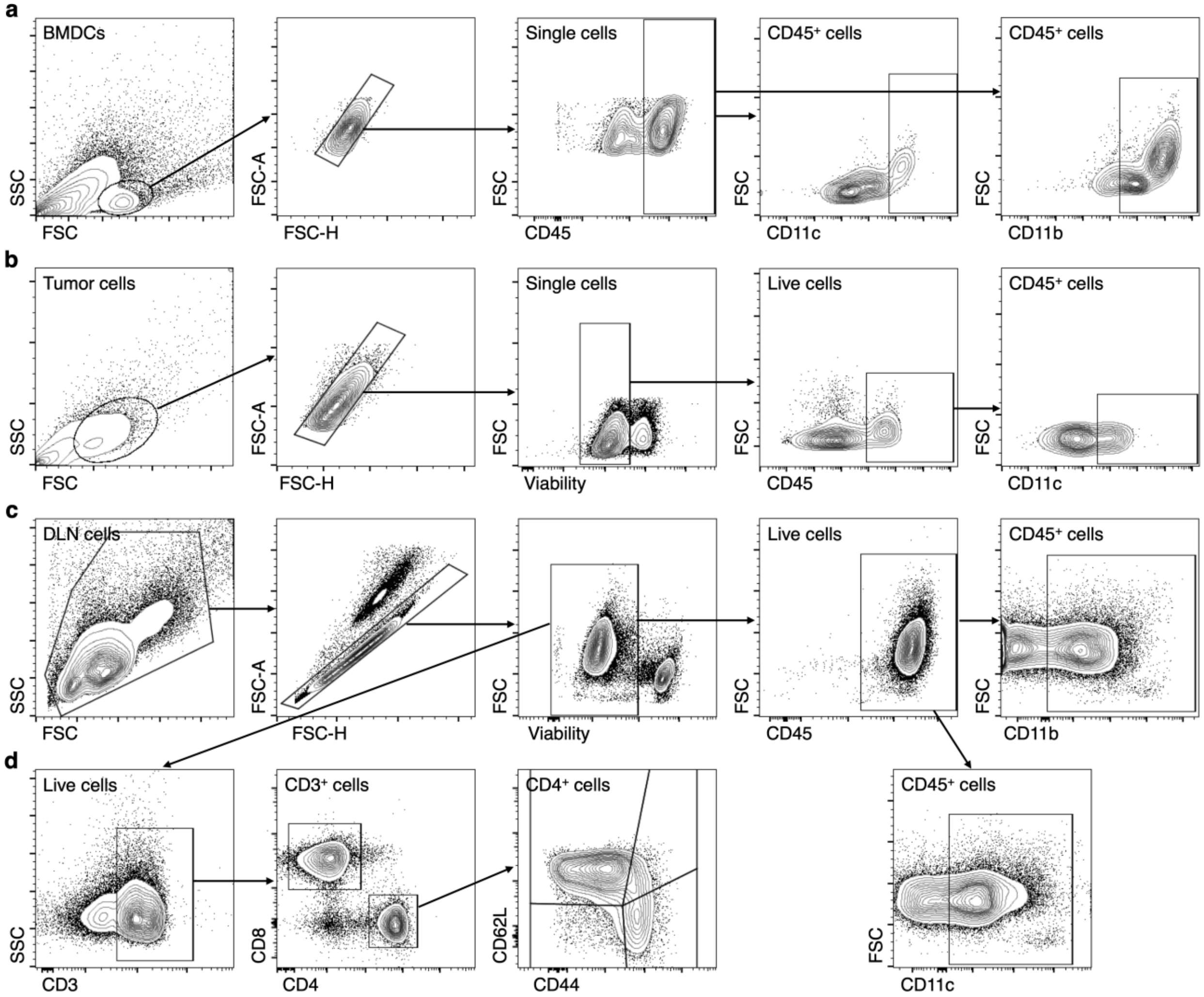

Supplementary Figure 7. a, Gating strategy to quantify BMDC maturation (Fig. 3c,d and Supplementary Fig. 2a,b) and to quantify nanoparticle binding and uptake with BMDCs in vitro (Fig. 3h,i). **b,** Gating strategy to quantify uptake of PNP in MC38 tumor tissue (Fig. 3j-l). **c,** Gating strategy to quantify the relative expression of MHC-II by cells in the DLN of mice (Fig. 5a). **d,** Gating strategy to determine percentage of $CD3^{+}$ immune cells, percentage of $CD8^{+}$ T cells, and proportion of $CD4^{+}$ T cells with various phenotypes in the DLN of mice (Fig. 5b-d).
