# Supplementary material for: Intratumoral immunotherapy using platelet-cloaked nanoparticles enhances antitumor immunity in solid tumors
Source: Nat Commun. 2021 Mar 31;12:1999. doi: 10.1038/s41467-021-22311-z (PMC8012593; doi:10.1038/s41467-021-22311-z)
Supplement: Supplementary file 3 — Reporting summary. [file 41467_2021_22311_MOESM3_ESM.pdf]

nature research

Corresponding author(s): Jie Zhang
Ronnie H. Fang

Last updated by author(s): Mar 4, 2021

Reporting Summary

Nature Research wishes to improve the reproducibility of the work that we publish. This form provides structure for consistency and transparency in reporting. For further information on Nature Research policies, see our Editorial Policies and the Editorial Policy Checklist.

Statistics

For all statistical analyses, confirm that the following items are present in the figure legend, table legend, main text, or Methods section.

n/a	Confirmed	
☐	☒	The exact sample size (*n*) for each experimental group/condition, given as a discrete number and unit of measurement
☐	☒	A statement on whether measurements were taken from distinct samples or whether the same sample was measured repeatedly
☐	☒	The statistical test(s) used AND whether they are one- or two-sided<br>*Only common tests should be described solely by name; describe more complex techniques in the Methods section.*
☐	☒	A description of all covariates tested
☐	☒	A description of any assumptions or corrections, such as tests of normality and adjustment for multiple comparisons
☐	☒	A full description of the statistical parameters including central tendency (e.g. means) or other basic estimates (e.g. regression coefficient) AND variation (e.g. standard deviation) or associated estimates of uncertainty (e.g. confidence intervals)
☐	☒	For null hypothesis testing, the test statistic (e.g. *F*, *t*, *r*) with confidence intervals, effect sizes, degrees of freedom and *P* value noted<br>*Give P values as exact values whenever suitable.*
☒	☐	For Bayesian analysis, information on the choice of priors and Markov chain Monte Carlo settings
☒	☐	For hierarchical and complex designs, identification of the appropriate level for tests and full reporting of outcomes
☒	☐	Estimates of effect sizes (e.g. Cohen's *d*, Pearson's *r*), indicating how they were calculated

Our web collection on statistics for biologists contains articles on many of the points above.

Software and code

Policy information about availability of computer code

Data collection	Flow cytometry data was collected on a Becton Dickinson Accuri C6 flow cytometer equipped with BD Accuri™ C6 Plus software and a Becton Dickinson FACSCanto II flow cytometer equipped with BD FACSDiva software. All the data was analyzed with Flowjo_V10 software. ELISA assay and HEK-Blue hTLR7 assay were detected using the SpectraMax M4 plate reader (Molecular Devices) with a multi-mode monochromator. UHPLC: UHPLC data was collected on the The Vanquish flex UHPLC (ThermoScientific) equipped with a diode array detector capable of pressures up to 12,000 psi. Nanoparticles were imaged using an FEI Tecnai Spirit G2 BioTWIN transmission electron microscope. Tumor tissue slides were imaged with a Hamamatsu Nanozoomer 2.0HT slide scanner. In vivo mice imaging performed using a Xenogen IVIS 200 with Living Image Software 3.0 (PerkinElmer). Hydrodynamic nanoparticle size and surface zeta potential were measured by dynamic light scattering using a Malvern Zetasizer Nano ZS.
Data analysis	Flow cytometry analysis was performed using Flowjo v10 (Becton, Dickinson & Company). Microscopy post-acquisition quantifications were made using QuPath v0.2.0 (open source software from Github). GraphPad Prism 8 software (GraphPad Software) was used for all statistical analysis. In vivo image analysis was performed using Xenogen Living Image Software 3.0.

For manuscripts utilizing custom algorithms or software that are central to the research but not yet described in published literature, software must be made available to editors and reviewers. We strongly encourage code deposition in a community repository (e.g. GitHub). See the Nature Research guidelines for submitting code & software for further information.

Data

Policy information about availability of data

All manuscripts must include a data availability statement. This statement should provide the following information, where applicable:

- Accession codes, unique identifiers, or web links for publicly available datasets
- A list of figures that have associated raw data
- A description of any restrictions on data availability

All data are available within the Article, Supplementary Information or available from the corresponding authors upon reasonable request. Source data are provided as a Source Data file.

Field-specific reporting

Please select the one below that is the best fit for your research. If you are not sure, read the appropriate sections before making your selection.

[x] Life sciences [ ] Behavioural & social sciences [ ] Ecological, evolutionary & environmental sciences

For a reference copy of the document with all sections, see nature.com/documents/nr-reporting-summary-flat.pdf

Life sciences study design

All studies must disclose on these points even when the disclosure is negative.

Sample size	Samples sizes were determined based on previous experience and by referencing previously published studies.
Data exclusions	No data were excluded from the analyses in this study.
Replication	All the experiments were replicated at least once (e.g. n greater than or equal to 2), with data shown being consistent and representative of two or more independent experiments.
Randomization	All In Vitro studies utilized homogeneous mixtures of cells which were allocated into various experimental groups and treated equally except for the imposed experimental variable as stated, thus no randomization was required for such experiments. For In Vivo studies, mice were randomized into treatment groups based on tumor volume in order to create experimental groups with approximately equal average tumor volumes at the initiation of treatment.
Blinding	No blinding was done since the same researchers performing the experiments were also responsible for planning the experiments and analysis of the data.

Reporting for specific materials, systems and methods

We require information from authors about some types of materials, experimental systems and methods used in many studies. Here, indicate whether each material, system or method listed is relevant to your study. If you are not sure if a list item applies to your research, read the appropriate section before selecting a response.

Materials & experimental systems

n/a	Involved in the study	
[ ]	[x]	Antibodies
[ ]	[x]	Eukaryotic cell lines
[x]	[ ]	Palaeontology and archaeology
[ ]	[x]	Animals and other organisms
[x]	[ ]	Human research participants
[x]	[ ]	Clinical data
[x]	[ ]	Dual use research of concern

Methods

n/a	Involved in the study	
[x]	[ ]	ChIP-seq
[ ]	[x]	Flow cytometry
[x]	[ ]	MRI-based neuroimaging

Antibodies

Antibodies used	FITC-conjugated annexin V (Biolegend), Alexa488-conjugated anti-human P-selectin (AK4; Biolegend), Alexa647-conjugated anti-human GPIbα (HIP1; Biolegend), Alexa647-conjugated anti-human αIIbβ3 (PAC-1; Biolegend), FITC-conjugated anti-mouse CD45 (30-F11; BD Biosciences), PE-conjugated anti-mouse CD80 (16-10A1; BD Biosciences), APC-conjugated anti-mouse CD86 (GL-1; Biolegend), FITC-conjugated anti-mouse CD45, PE-conjugated anti-mouse CD11b (M1/70; Biolegend), PE/Cy7-conjugated anti-mouse CD11c (N418; Biolegend), BV510-conjugated anti-mouse CD3 (17A2; Biolegend), FITC-conjugated anti-mouse CD4 (RM4-5; eBiosciences), APC/Cy7-conjugated anti-mouse CD8 (53-6.7; Invitrogen), PerCP/Cy5.5-conjugated anti-mouse CD62L (MEL-14; eBioscience), APC-conjugated anti-mouse CD44 (IM7; BD Biosciences), V500-conjugated anti-mouse CD45 (30-F11; BD Biosciences),

	APC-conjugated anti-mouse MHC-II (M5/114.15.2; Tonbo Bioscience), APC/Cy7-conjugated anti-mouse CD11b (M1/70; BD Biosciences), PE/Cy7-conjugated anti-mouse CD11c.
Validation	We relied on the validation process performed by the manufacturers, which are available on the manufacturer's website for each respective antibody.

Eukaryotic cell lines

Policy information about cell lines

Cell line source(s)	HEK-Blue hTLR7 reporter cells (Invivogen), HEK-Blue hTLR8 reporter cells (Invivogen), MC38 murine colon adenocarcinoma cells (Kerafast), MDA-MB-231 human mammary gland adenocarcinoma cells (HTB-26; American Type Culture Collection), 4T1 murine mammary gland cancer cells (CRL-2539; American Type Culture Collection), HT-29 human colorectal adenocarcinoma cells (HTB-38; American Type Culture Collection)
Authentication	None of the cell lines used were authenticated.
Mycoplasma contamination	All cell lines tested negative for contamination with mycoplasma.
Commonly misidentified lines (See ICLAC register)	No commonly misidentified cell lines were used in this study.

Animals and other organisms

Policy information about studies involving animals; ARRIVE guidelines recommended for reporting animal research

Laboratory animals	Mice, wild-type C57BL/6 and BALB/c, female, 6 weeks old, Charles River Labs. Animals were maintained in standard housing at 68-75 degrees Farenheit, 40-60% relative humidity, and 12 h light/dark cycles.
Wild animals	No wild animals were used in this study
Field-collected samples	This study did not involve samples collected from the field.
Ethics oversight	All animal experiments were approved by the Institutional Animal Care and Use Committee (IACUC) of University of California, San Diego (UCSD).

Note that full information on the approval of the study protocol must also be provided in the manuscript.

Flow Cytometry

Plots

Confirm that:

- [ ] The axis labels state the marker and fluorochrome used (e.g. CD4-FITC).
- [x] The axis scales are clearly visible. Include numbers along axes only for bottom left plot of group (a 'group' is an analysis of identical markers).
- [x] All plots are contour plots with outliers or pseudocolor plots.
- [ ] A numerical value for number of cells or percentage (with statistics) is provided.

Methodology

Sample preparation	The inguinal draining lymph node (DLN) (on the same side as the tumor) was processed into a single cell suspension by shearing the tissue using a 50-µm cell strainer.
Instrument	A Becton Dickinson Accuri C6 flow cytometer (BD Biosciences) and a FACSCanto II (BD Biosciences).
Software	BD Accuri™ C6 Plus software, BD FACSDiva software, Flowjo_V10 software
Cell population abundance	Sorting was not used in this study.
Gating strategy	The lymphocytes were gated on by plotting SSC vs. FSC. The single cells were selected by plotting FSC-A vs. FSC-H, followed by selection of live cells and CD45+ cells.

- [x] Tick this box to confirm that a figure exemplifying the gating strategy is provided in the Supplementary Information.
